# Editorial: Crosstalk between intonation and lexical tones: Linguistic, cognitive and neuroscience perspectives

**DOI:** 10.3389/fpsyg.2022.1101499

**Published:** 2022-12-08

**Authors:** Hatice Zora, Carlos Gussenhoven, Annie Tremblay, Fang Liu

**Affiliations:** ^1^Department of Neurobiology of Language, Max Planck Institute for Psycholinguistics, Nijmegen, Netherlands; ^2^Department of Foreign Languages and Literatures, National Yang Ming Chiao Tung University, Hsinchu, Taiwan; ^3^Department of Language and Communication, Radboud University, Nijmegen, Netherlands; ^4^Department of Linguistics, University of Kansas, Lawrence, KS, United States; ^5^Department of Languages and Linguistics, The University of Texas at El Paso, El Paso, TX, United States; ^6^School of Psychology and Clinical Language Sciences, University of Reading, Reading, United Kingdom

**Keywords:** prosody, intonation, lexical tone, categorical pitch, gradient pitch

The interplay between categorical and continuous aspects of the speech signal remains central and yet controversial in the fields of phonetics and phonology. The division between phonological abstractions and phonetic variations has been particularly relevant to the unraveling of diverse communicative functions of pitch in the domain of prosody. Pitch influences vocal communication in two major but fundamentally different ways, and lexical and intonational tones exquisitely capture these functions. Lexical tone contrasts convey lexical meanings as well as derivational meanings at the word level and are grammatically encoded as discrete structures. Intonational tones, on the other hand, signal post-lexical meanings at the phrasal level and typically allow gradient pragmatic variations. Since categorical and gradient uses of pitch are ubiquitous and closely intertwined in their physiological and psychological processes, further research is warranted for a more detailed understanding of their structural and functional characterisations. This Research Topic addresses this matter from a wide range of perspectives, including first and second language acquisition, speech production and perception, structural and functional diversity, and working with distinct languages and experimental measures. In the following, we provide a short overview of the contributions submitted to this topic.

## Behavioral investigation of tonal and intonational categoriality

Two original research articles addressed the categoriality debate of tones by expanding on existing behavioral measures. Using a Sequence Recall Task (SRT), Gussenhoven et al. tested whether a high performance in SRT indicates the lexical status of tonal information in a similar fashion to word stress. Data from speakers of non-tonal, semi-tonal, and tonal languages indicated that a tonal SRT is unlikely to discriminate between tonal and non-tonal languages due to the rich phonological nature of tone, and a number of factors affected performance, like the phonetic salience of a pitch contrast and the complexity of a language's tone system.

Rodd and Chen investigated the question whether intonation events have a categorical mental representation similar to those of segments and lexical tones by testing for a Perceptual Magnet Effect (PME). Perceived goodness and discriminability of re-synthesized productions of a Dutch rising pitch accent were evaluated by Dutch listeners. The results provided evidence for categoricalness of pitch accents, however yielding a weaker and more transient PME in pitch accents compared to the PME in lexical tones and phonemes.

## Phonetic correlates of interaction between tonal functions

Phonetic correlates of interaction between lexical tone and intonation were examined by two original research articles. Zhang et al. explored how citation and neutral tones affect the perception of intonation in Mandarin. Listeners determined whether disyllabic words with citation and neutral tones formed a question or statement. The results indicated that the phonetic realizations of the neutral tone and of citation tones, realized with diverse pitch ranges and levels, determine intonation perception.

Wang et al. investigated the interaction between informative and articulatory pitch control, and specifically studied *downstep* in Mandarin and its interaction with focus and phrasing. Tonal environment, boundary strength, and focus were systematically manipulated in a production experiment. The results showed that intonation was shaped by both informative functions and articulatory constraints; downstep seems to be a phonetic effect and the interaction between focus and downstep is gradual.

## Crosstalk between tone and intonation from the perspective of second language acquisition

Two original research articles studied the multifaceted function of tone from the perspective of second language (L2) acquisition. Using SRT, Kim and Tremblay examined whether listeners' use of intonational cues to a segmental contrast in the native language (L1) can facilitate the processing of an intonationally cued lexical stress contrast in the L2 by comparing Seoul Korean and French L2 learners of English. Korean listeners, who can use intonational cues to perceive segmental contrasts in their L1, outperformed French listeners, for whom segmental contrasts are not cued by intonational cues in the L1. These results suggest that cues can transfer across different types of linguistic contrasts.

Zahner-Ritter et al. used an imitation paradigm to investigate how L2 learners with a tone language as their L1 acquire pitch accents in an L2 intonation language. The authors tested the ability of Mandarin and Italian learners to imitate intonational pitch accents in German. The results indicated that experience with a tone language yields neither an advantage nor a disadvantage in the acquisition of L2 intonational pitch accents. The findings revealed instead a general cross-linguistic influence in the realization of pitch contrasts as well as improvement with higher proficiency.

## Neuroscienctific evidence for structural and functional specialization of tones

Two contributions to this issue, one original research article and one hypothesis and theory article, explored the structural and functional specialization of tones using neuroscientific paradigms. Wei et al. investigated the hemispheric specialization of Chinese linguistic tones using magnetic resonance imaging and electroencephalography recordings by comparing patients with a stroke in the right temporal lobe and healthy subjects. The brain responses were lateralized in the left hemisphere for stroke patients, and in the right hemisphere for healthy individuals, indicating that the right temporal lobe is a core area for tonal processing.

Roll addressed the questionable function of the Swedish word accent contrast. Given that the two word accents do not mainly serve a lexical contrast function, they have a very low functional load from a traditional phonological contrast perspective. However, based on psychological and neurophysiological evidence and a novel analysis, the author proposes that the chief function of Swedish word accents is to predict upcoming morphological structures and facilitate lexical processing rather than being lexically distinctive.

## Infant acquisition of linguistic and paralinguistic functions of pitch

The developmental course of pitch discrimination was addressed in a review article. Liu et al. discussed the lexical, intonational, and emotional functions carried by pitch, tracking how they are acquired throughout infancy. Based on a review of empirical evidence and theoretical considerations, the authors propose the Learnability Hypothesis, according to which the diverse functions of pitch are distinguished and acquired through native/environmental experiences.

## Conclusion

Altogether, the articles in this Research Topic provide us with valuable information on the human disposition for stability and variability in communication, give us new insights into how (para)linguistic expressivity is built through pitch modulations, and establish new directions for future research. Key themes for further investigations include theoretical and neural network models of the interplay and integration of different tonal functions as well as a closer examination of tonal and non-tonal varieties of the same language.

## Author contributions

All authors listed have made a substantial, direct, and intellectual contribution to the work and approved it for publication.

